# A novel loop-mediated isothermal amplification-based test for detecting *Neospora caninum* DNA

**DOI:** 10.1186/s13071-017-2549-y

**Published:** 2017-11-29

**Authors:** Andrea Estefanía Ramos, Marina Muñoz, Jesús Alfredo Cortés-Vecino, Paola Barato, Manuel Alfonso Patarroyo

**Affiliations:** 1 Molecular Biology and Immunology Department, Fundación Instituto de Inmunología de Colombia, Bogotá, Colombia; 2 Veterinary Medicine and Zootech Faculty, Universidad Nacional de Colombia, Bogotá, Colombia; 3 Corporación Patología Veterinaria (Corpavet), Bogotá, Colombia; 4 School of Medicine and Health Sciences, Universidad del Rosario, Bogotá, Colombia

**Keywords:** *Neospora caninum*, Neosporosis, Loop-mediated isothermal amplification, Semi-nested PCR, *Nc-5* gene

## Abstract

**Background:**

*Neospora caninum* is a cyst-forming, coccidian parasite which is known to cause neurological disorders in dogs and abortion and neonatal mortality in cows and other livestock. This study reports the development of a loop-mediated isothermal amplification (LAMP) assay based on the *Neospora caninum Nc-5* gene and compares its efficacy for detecting DNA to that of a semi-nested PCR test.

**Results:**

Six primers were designed based on the *Nc-5* repeat region of *N. caninum*. Specific LAMP primers led to successful amplification of *N. caninum* DNA at 63 °C in 30 min. The LAMP assay was highly specific (i.e. it did not reveal cross-reactivity with other parasite species) and had a low *N. caninum* plasmid DNA limit of detection (1 fg), which is ten times higher than that for the semi-nested PCR. LAMP applicability was evaluated using a set of naturally-infected samples (59 from canine faeces and five from bovine abortions). Thirty-nine percent (25/64) of the naturally-infected samples were positive for *N. caninum* DNA by LAMP and 36% (23/64) by semi-nested PCR. However, the LAMP assay is much faster to perform than semi-nested PCR and provides results in 30 min.

**Conclusion:**

The optimized reaction conditions described in this study resulted in a sensitive, specific and rapid technique for detecting *N. caninum* DNA. Considering the advantages of LAMP for detecting *N. caninum* DNA, further assays aimed at testing its usefulness on a wider range of field samples are recommended.

**Electronic supplementary material:**

The online version of this article (doi: 10.1186/s13071-017-2549-y) contains supplementary material, which is available to authorized users.

## Background

*Neospora caninum* is an obligate intracellular tissue protozoan parasite belonging to the Phylum Apicomplexa (subclass: Coccidia) [1]. The infectious disease caused by this parasite is called neosporosis and is mainly associated with severe neuromuscular disease in dogs and other canids (considered definitive host) and abortion in cows (the main intermediate host) and other livestock [2–6], causing major economic losses for livestock farmers [7]. The role of domestic dogs and other canids is essential for the life-cycle’s continuity due to the excretion of environment-resistant oocysts, this being the only form of horizontal transmission in herbivores [8]. The other way of acquiring the infection is by consuming oocysts from aborted tissue (exogenous origin) or during the birth of a persistently-infected individual (endogenous origin) [7].

The most common techniques for diagnosing *N. caninum* are those aimed at detecting specific antibodies in sera; different assays have been developed including the indirect fluorescent antibody test (IFAT), various enzyme-linked immunosorbent assays (ELISA), immunoblotting (IB) and direct agglutination tests (DAT) [9, 10]. The advantage of serological tests is that they provide information regarding infection stage [11]; however, specific antibodies’ fluctuation during infection limits serological methods because such fluctuation sometimes comes below serological tests’ detection limits [12]. Immunohistochemical staining was one of the first techniques used for diagnosing *N. caninum* infection; this technique is often used for demonstrating the parasite’s presence in lesions, particularly in the brain and heart [13]. Nevertheless, immunohistochemistry techniques have been shown to have relatively low sensitivity, and this may be related to low parasite number and the degree of autolysis in analyzed tissues [10]. Regarding molecular diagnosis of infection, polymerase chain reaction (PCR)-based tests have the advantage of being able to amplify small amounts of parasite DNA in different types of biological samples and are directed towards amplifying different genes, the *N. caninum Nc-5* repeat sequence and internal transcribed spacer (ITS1) being most frequently used [14]. Different PCR formats have been developed recently for increasing sensitivity in detecting *N. caninum* DNA, i.e. nested, semi-nested, and real-time PCR [9]. However, PCR diagnosis implies the use of sophisticated equipment such as a thermocycler for amplifying nucleic acids, as well as requiring personnel trained in such area and additional time for detecting the amplified products [15].

Since loop-mediated isothermal amplification (LAMP) was introduced in 2000, this technique has represented a cost-effective alternative for molecular diagnosis [16]. LAMP allows the amplification of nucleic acids; it provides high specificity, sensitivity, and rapidity in isothermal conditions and is based on the strand displacement reaction principle and the formation of loop structures amplifying a sequence of interest. This involves using a polymerase having strand displacement activity and six primers specifically recognising eight different regions in the target sequence [17].

Unlike conventional PCR, LAMP is carried out in isothermal conditions between 60 and 65 °C, thereby eliminating the need for using a thermocycler. LAMP has been widely used in detecting infections caused by different microorganisms, including those caused by protozoan parasites such as *Toxoplasma gondii* [18–20], *Trypanosoma* spp. [21], *Theileria annulata* [22], *Babesia caballi* [23] and *Leishmania* spp. [24, 25]. This work was thus aimed at describing the development of a LAMP-type test for detecting *N. caninum* infection and comparing its detection limit to that of the previously-reported semi-nested PCR detection method [26].

## Methods

### Designing primers

Six primers were designed based on the single *N. caninum Nc-5* repeat region (GenBank: AY459289.1) using LAMP primer design software (Primer Explorer V4) (https://primerexplorer.jp/e/). The two outer primers, called forward (F3) and backward (B3), allow strand displacement; the inner primers, forward inner (FIP) and backward inner primers (BIP), have two types of sequences: the sense and antisense sequences of the region to be amplified. One is for priming during the non-cyclic step and the other for self-priming during cyclic amplification. FIP contains the F1C region (complementary to the F1 region) and the F2 region; BIP contains the B1C sequence (complementary to B1) and the B2 sequence [17]. The design of loop primers (FLP and BLP) is also included for accelerating the reaction and reducing amplification time [27]. The loop primers correspond to regions between F1 and F2 and B1 and B2.

LAMP test selectivity was determined by in silico analysis using *Nc-5* gene DNA sequences from different isolates, taking the information currently available in databases into account. Homologous sequences were determined using the BLAST tool which is available on the National Center for Biotechnology Information (NCBI) page, taking the sequence for the *Nc-5* gene used for designing the primers as the query sequence.

### Preparing plasmid DNA

A 350 base pair (bp) fragment from the *Nc-5* region was amplified from *N. caninum* Bahía strain total DNA using high fidelity KAPA HiFi HotStart DNA polymerase **(**KAPA Biosystems, Woburn, MA, USA) and a previously reported set of primers: Np21+ (5′-CCC AGT GCG TCC AAT CCT GTA AC-3′) / Np6+ (5′-CTC GCC AGT CAA CCT ACG TCT TCT-3′) [28]. The amplified product was ligated into the pGEM-T easy vector (Promega, Madison, WI, USA) multiple cloning site and then used for transforming *E. coli* JM109 competent cells (Promega), following the manufacturer’s recommendations. Extracted plasmid DNA was confirmed by sequencing and used as amplification template in all LAMP standardization assays.

### LAMP reaction

Bst 2.0 DNA Polymerase (New England Biolabs, Herts, UK), having strand displacement activity, was used for LAMP assay amplification at 25 μl final reaction volume. Optimization of LAMP assay conditions was carried out through evaluation of reagents concentrations following ranges reported in the literature for pathogen detection [29, 30] and the manufacturer’s instructions. During this optimization process different concentrations from 1 to 1.6 M of betaine (Sigma-Aldrich, St. Louis, MO, USA), 1.4 mM to 2.8 mM of dNTP’s (Bioline, UK) and from 4 to 8 mM MgSO_4_ [31] were tested in PCR master mix. Various amounts of primers (3 and 5 pmol F3 and B3; 30 and 40 pmol FIP and BIP; and 5, 10, and 15 pmol FLP and BLP) were used for identifying the best conditions. The reaction mixture was incubated at 61 °C, 63 °C or 65 °C for 40, 50 or 60 min, for determining optimum LAMP assay temperature and incubation time. The reaction was stopped by heating at 80 °C for 10 min, according to the manufacturer’s recommendations. The best reaction conditions were defined as those where the generation of the stem-loop structure into LAMP mixture allow the production of an amplification pattern of high quality (defined bands inter-spaciads, ladder-like banding) [32]. The optimal reaction mix was established as follows: 1× isothermal amplification buffer (20 mM Tris-HCl, 50 mM KCl, 10 mM (NH_4_)_2_SO_4_, 2 mM MgSO_4_, 0.1% Tween-20), 2.8 mM of each dNTP, 1.6 M betaine (Sigma-Aldrich), 8 U Bst DNA polymerase and 1 ng of plasmid DNA. All reagents and samples were mixed on ice. An additional reaction with molecular grade water instead of template DNA was included as a negative control due to the LAMP reaction’s high sensitivity in each run, and the following precautions were taken: manipulating the reaction tubes was minimised, and the LAMP assay’s different steps were carried out in separated environments to prevent cross-contamination. The amplification products were submitted to agarose gel electrophoresis analysis using 2% agarose and stained with SYBRsafe (Invitrogen Corp., California, CA, USA) in an independent area.

### LAMP analytical performance

**Fig. 1:**
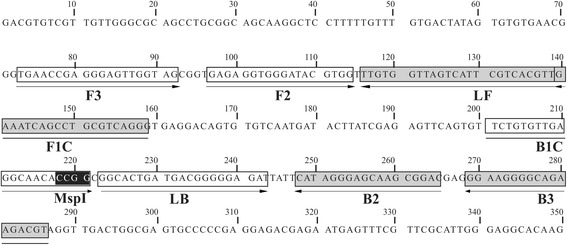
Details of the LAMP assay primer sets used for amplifying *Neospora caninum*. The location of the primers used in LAMP concerning *Nc-5*. The sequence in the black box represents the location of the *Msp*I restriction enzyme site

Three different assays were used for evaluating LAMP test analytical performance. DNA from 5 closely-related protozoan parasites (*Sarcocystis cruzi*, *Sarcocystis hominis*, *Hammondia hammondi*, *Toxoplasma gondii* and *Cryptosporidium parvum*) was used as a template for evaluating LAMP test selectivity. *Sarcocystis cruzi*, *S. hominis* and *H. hammondi* DNA controls were kindly donated by the United States Department of Agriculture (USDA, Beltsville). *Toxoplasma gondii* DNA came from the ME49 strain (clonal type II), cat 19, collected during June 2014, donated by Professor Jitender Dubey (USDA, Beltsville) and the *C. parvum* control was bought from the University of Texas. Two different concentrations were used as a template when evaluating independent reactions for each species: direct DNA extraction and at 1:10 concentration (i.e. 20–40 ng, the range equivalent to the amount of DNA used for any PCR assay) [33]. Two μl of DNA from each species at the following concentrations: 19 ng/μl *S. cruzi*, 10 ng/μl *S. hominis*, 175 ng/μl *H. hammondi*, 11 ng/μl *T. gondii* and 90 ng/μl *C. parvum* were used as template in the initial test. The second verification step involved extracting DNA from healthy domestic dogs’ faecal samples and fetal brain tissue negative for *Neospora*. Such DNA was used for evaluating the absence of primer cross-reactivity with hosts’ DNA. A third verification step involved evaluating the sequence of LAMP products digested with the *Msp*I restriction enzyme (16 h incubation at 37 °C), according to the manufacturer’s instructions. The resulting fragments were analyzed by electrophoresis. The *Msp*I enzyme has a recognition site in the region between B1C and B2 (Fig. 1a), meaning that it would be expected that 218 and 132 bp fragments would be obtained after treating the product resulting from LAMP amplification.

The limit of detection (LoD) was determined following the methodology reported earlier for LAMP tests [34, 35]. Serial 10-fold plasmid DNA dilutions (ranging from 1 × 10^−1^ to 1 × 10^−6^ ng) were made in H_2_O. LAMP assay LoD was compared to that for semi-nested PCR. Each assay was done in triplicate and incubated at 63 °C for 30 min. Semi-nested PCR assays were carried out using previously reported primers [26]. All PCR reactions were performed in a 10 μl volume containing: 1× NH_4_ reaction buffer, 0.5 μM each primer, 2.5 mM MgCl_2_, 0.2 mM each dNTP and 0.5 U Biolase DNA polymerase (Bioline). The first amplification round followed the originally reported conditions [26]; the second involved using a microliter of first PCR product for amplification with the internal semi-nested primers in the following conditions: 95 °C for 5 min and then 35 cycles at 95 °C for 15 s, 54 °C for 30 s, 72 °C for 1 min, followed by a final extension step at 72 °C for 10 min. An aliquot of each PCR product was examined on 2% agarose gel stained with SYBRsafe.

The analytical performance of both tests was also evaluated directly using *N. caninum* genomic DNA (NC-1 strain); LoD was determined using 10-fold serial dilutions ranging from 1 × 10^−1^ (0.5 ng/μl) to 1 × 10^−6^ (5 fg/μl) subjected to amplification in the conditions mentioned above.

### LAMP assay for *N. caninum* with clinical samples

A set of 64 samples was used for evaluating the LAMP method regarding *N. caninum* detection. The set consisted of 3 brain samples from bovine abortions, 2 samples from the pool of bovine abortion tissue (lungs, heart, liver and spleen) and 59 samples of canine faeces (taken from around cattle herds having a history of abortion). The pool of bovine abortion tissue came from abortions of cows positive for *N. caninum* by IFA. Such tissue was evaluated by histopathology; this led to lesions compatible with neosporosis being identified. A Wizard Genomic DNA Purification Kit (Promega) was used for extracting DNA from clinical abortion tissue samples and a Stool DNA Isolation Kit (Norgen Biotek Corp., Thorold, ON, Canada) for extracting DNA from samples of faeces, following the manufacturers’ instructions in both cases. The 64 samples were used for *N. caninum* infection identification, using both the LAMP method and the previously-described semi-nested PCR method [26]. Both molecular assays were carried out independently in double-blind experiments; their results were reported regarding *N. caninum* infection frequency. Agreement between both molecular assays regarding *N. caninum* DNA detection was evaluated as an indicator of LAMP method performance, using the kappa (κ) coefficient. All results were reported along with their 95% confidence intervals (95% CI) which were calculated using the bootstrap method. Stata 10 software was used for statistical analysis.

## Results

### Selection of designed primers

Table 1 provides primer sequences and Fig. 1 their location in the *Nc-5* sequence. LAMP test selectivity was evaluated by in silico analysis, comparing reported sequences. Eighty-three *Nc-5* gene sequences from different isolates were recovered from databases and aligned to check how conserved the primers designed for the LAMP method were; 38 sequences were excluded from further analysis as they did not have the required length for bioinformatics analysis (i.e. they did not cover Nc-F3 and Nc-B3 primers’ annealing sites). A further group of 9 sequences was excluded as lacking clinical relevance, since the host from which they came did not play a relevant role in *N. caninum*’s biological cycle and thus isolates infecting these species lacked epidemiological interest, leaving 36 sequences to be analyzed (information regarding all the sequences found and describes the exclusion scheme is provided in Additional file 1: Table S1).

**Table 1:** A list of primers used for the LAMP method

Primer name	Type	Length	Localization^a^	Sequence (5′–3′)
F3	Forward outer	20	73–92	TGAACCGAGGGAGTTGGTAG
B3	Reverse outer	18	269–286	ACGTCTTCTGCCCCTTCC
FIP^b^	Forward inner	40	F1C, 140–160	ACCCTGACGCAGGCTGATTTC
	(FIP = F1C + F2)		F2, 97–115	GAGAGGTGGGATACGTGGT
BIP^b^	Reverse inner	40	B1C, 200–221	TTCTGTGTTGAGGCAACACCGG
	(BIP = B1C + B2)		B2, 248–265	GTCCGCTTGCTCCCTATG
FLP	Forward Loop	24	116–139	AACGTGACGAATGACTAACCACAA
BLP	Reverse Loop	21	223–243	GGCACTGATGACGGGGGAGAT

^a^Localization of the primers is based on the nucleotide sequence of *N. caninum* Nc-5 unique repetitive region (GenBank: AY459289.1)

^b^Each inner primer of LAMP contains two connected primers

Multiple sequence comparison by log-expectation (MUSCLE) [36] was used for comparing the set of sequences selected for analysis; the sequences’ overall identity was 84.72%. Regarding the primers’ annealing sites, 90.0 and 100% identity, respectively, was found at the F3 and B3 primer annealing sites. Differences in primer F3 annealing site were only observed in 4 sequences which had two varying positions. Additional file 2: Figure S1 shows the annealing sites for all LAMP-designed primers, as well as the identity percentage for all sequences analyzed. The recognition sites for the primers designed for the LAMP method were conserved in most *N. caninum* isolates and the strains analyzed.

### LAMP optimization

**Fig. 2:**
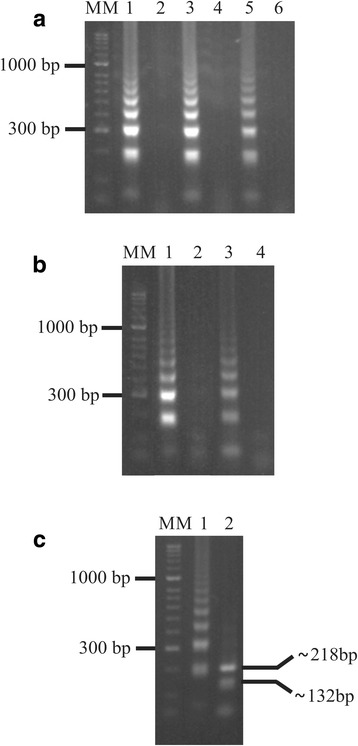
Using the LAMP assay for detecting *N. caninum* plasmid DNA containing the *Nc-5* region on a SYBRsafe stained agarose gel. **a** The effects of reaction time. Lanes 1 and 2: 60 min; Lanes 3 and 4: 50 min; Lanes 5 and 6: 40 min. Lanes 2, 4 and 6 represent negative controls for each reaction time. **b** Optimizing LAMP assay conditions. Lanes 1 and 2: 30 min; Lanes 3 and 4: 20 min. Lanes 2 and 4 are the negative control for each reaction time. **c** Restriction analysis of *N. caninum* LAMP products amplified from plasmid DNA containing the *Nc-5* region. The digestion products were run on a 3% agarose gel. Lane 1: *N. caninum* LAMP product; Lane 2: *Msp*I digestion of *N. caninum* product (132–218 bp bands, according to predicted size). Lane MM in **a**, **b** and **c** is the HyperLadder II (Bioline) DNA molecular marker

It was found that the optimum concentrations for the primers in the LAMP reaction were 40, 5 and 15 pmol for the inner/outer/loop primers, respectively. Using specific primers for LAMP resulted in successful amplification of *N. caninum* plasmid DNA from 61 °C to 65 °C; 63 °C was selected as optimum temperature for the LAMP assay as a defined ladder-type band pattern was identified on agarose gel according to that expected for this type of experiment (detected after 40, 50 and 60 min reaction time had elapsed) (Fig. 2a). The preceding was due to the formation of a mixture of various sized stem-loop DNA and cauliflower-like structures from the alignment between the alternately inverted repeats in the target sequence in the same chain [17]. Two shorter reaction times were evaluated (20 and 30 min) for determining the minimum reaction time in which *N. caninum* DNA could be amplified; it was found that a well-defined and expected ladder-type pattern was obtained after 30 min and thus chosen as the reaction time for the method (Fig. 2b). The assay described above provided an alternative technique for detecting *N. caninum* DNA rapidly, since including loop primers in the reaction led to reducing DNA amplification time (usually taking 60 min), amplifying 1 ng *N. caninum* DNA at 63 °C.

### Analysis of LAMP test performance

LAMP test selectivity was successfully demonstrated by the absence of amplification when DNA from other related parasites (*S. cruzi*, *S. hominis*, *H. hammondi*, *T. gondii* and *C. parvum*) and later when DNA extracted from healthy domestic dogs’ fecal samples and fetal brain tissue negative for *Neospora* was used as amplification template, during a 60 min LAMP reaction (data not shown). The amplified product restriction pattern was that to be expected following Msp I digestion (Fig. 2c). These findings confirmed the optimal performance based on the single *Nc-5* sequence in the *N. caninum* genome used in this study, thereby ensuring selectivity in amplifying this parasite’s DNA.

**Fig. 3:**
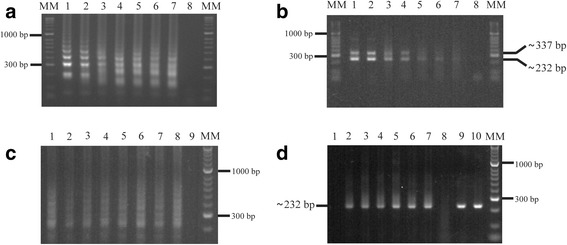
LAMP and semi-nested PCR Limit of Detection (LoD). **a** LAMP reaction. **b** Semi-nested PCR. Ten-fold serial dilutions of plasmid DNA were used in both **a** and **b**; they contained the *Nc-5* region for detecting *N. caninum* by agarose gel electrophoresis analysis. From left to right: Lane MM: HyperLadder II (Bioline) DNA molecular marker; Lane 1: amplification of 1 ng plasmid DNA containing the cloned *N. caninum* fragment; Lanes 2–7: 10-fold serial dilutions of *N. caninum* plasmid DNA (10^−1^ to 10^−6^ ng); Lane 8: negative control without target DNA. Semi-nested PCR products showed specific amplification of *N. caninum*, having 10^−5^ ng LoD whereas LAMP LoD was 10^−6^ ng. LAMP and semi-nested PCR LoD in both **c** and **d** using serial dilutions of *N.caninum* genomic DNA (NC-1 strain) by visualization on an agarose gel. **c** LAMP reaction. Lane MM: HyperLadder II (Bioline) DNA molecular marker; Lane 1: amplification of *N. caninum* genomic DNA (50 ng); Lanes 2–7: 10-fold serial dilutions (10^−1^ to 10^−6^ ng); Lane 8: positive control (plasmid DNA); Lane 9: negative control (no DNA template). **d** Semi-nested PCR. Lane MM: HyperLadder II (Bioline) DNA molecular marker; Lane 1: negative control (no DNA template); Lane 2: amplification of *N. caninum* genomic DNA (50 ng); Lanes 3–8: 10-fold serial dilutions (10^−1^ to 10^−6^ ng); Lanes 9, 10: positive controls (plasmid DNA)

The LoD for the products amplified in the LAMP assay (Fig. 3a) and the semi-nested PCR (Fig. 3b) was 1 fg and 10 fg for *N. caninum* plasmid DNA. These results were consistent when *N. caninum* genomic DNA LoD was determined directly; LAMP assay LoD was 5 fg (Fig. 3c), while semi-nested PCR LoD was 50 fg (Fig. 3d). The assay time used for determining LoD was 30 min.

### Using the LAMP assay with clinical samples

A set of samples including 5 fetal tissues from bovine abortions and 59 samples of canine faeces was tested by LAMP and compared to the semi-nested PCR method (Table 1). *N. caninum* infection frequency detected by the LAMP method was 39.1% (*n* = 25; 95% CI: 27.10–52.0%], this being higher than that detected by semi-nested PCR (35.9%) [*n* = 23; 95% CI: 24.31–48.90%). Comparative analysis of the techniques’ results gave a discordant result for the 14 samples (6 were only positive for semi-nested PCR and 8 just for the LAMP test). Overall agreement between both molecular assays was 78.13%, kappa coefficient being 0.533 (95% CI: 0.263–0.721); such estimator came within the “moderate” range according to Remmerbach’s classification [37].

## Discussion

Despite the importance of *N. caninum* in veterinary medicine (i.e. recognized as a leading cause of infectious abortions in cattle worldwide), no fully effective vaccine or treatment is available to prevent or cure such infection [38]. The development of DNA-based methods such as conventional PCR [39, 40], nested and semi-nested PCR [41, 42] and different quantitative PCR types [43, 44] have contributed towards diagnosing *N. caninum* infection. However, some of these methods are time-consuming, laborious and may require the use of specialized equipment. More recently, the LAMP technique for DNA amplification [17] has been shown to be versatile and efficient, being applied for detecting DNA from different microorganisms [45]. Nonetheless, there have been no reports regarding LAMP being used for detecting *N. caninum.* The LAMP method established in this study can detect *N. caninum* DNA in isothermal conditions at 63 °C in just 30 min. Compared to semi-nested PCR (requiring three primers and two amplification rounds in a thermocycler, results being obtained in 2–4 h), LAMP saves time, is easily carried out and requires no special equipment. It was thus proposed that the LAMP method represent a species-specific assay for detecting *N. caninum* DNA as the expected products were produced at the predicted band sizes, following LAMP-amplified product digestion by restriction enzymes. The LAMP technique’s specificity was due to using six primers in the reaction which recognized eight different regions in the DNA sequence of interest [27]. LAMP assay gave amplification at a 10-fold lower level, suggesting that LAMP was more sensitive than semi-nested PCR.

It was found that 25 of 64 samples (39.1%) were positive for *N. caninum* by LAMP analysis, whereas 23 of 64 samples (35.94%) were positive by semi-nested PCR. Even though both molecular assays revealed relatively similar infection frequencies, the LAMP method detected a slight increase in infection frequency which might have been due to the higher sensitivity reported for this method compared to that of the nested PCR [46, 47], as shown for *N. caninum* by the analytical tests described above.

Our results showed LAMP’s potential use in detecting DNA in environmental samples contaminated with *N. caninum* DNA such as the faeces of infected dogs which can be attributed to oocyst excretion or the result from *N. caninum*-infected tissue ingested by dogs. The high *N. caninum* infection frequency found in samples of dog faeces agreed with other studies describing infection caused by *N. caninum* being higher in rural dogs, probably related to the availability of cattle carcasses and abortion products in such areas [48]. Despite 17 samples being positive by both techniques, there were a few discordant results regarding LAMP and semi-nested PCR (Table 1). The eight canine faeces samples positive by LAMP but negative by semi-nested PCR may have had few parasite DNA molecules below the semi-nested PCR analysis detection limit. Similar results have been described by other authors where higher sensitivity has been reported for LAMP, particularly when DNA has been isolated from veterinary or environmental samples [46, 49, 50]. Variation regarding DNA detection by LAMP has been reported when comparing different kits’ performance when using faecal samples [51]. It should be noted that the high LAMP sensitivity level could lead to non-specific amplification [52]; it has been reported that is better to assess DNA amplification by the naked eye using hydroxynaphthol blue (HNB) or calcein (due to precipitate generation) [32], rather than by gel electrophoresis, thus avoiding the tube to be opened after the reaction has been completed [53]. On the other hand, it has been described that using impure DNA or partially purified DNA as the template could inhibit or affect LAMP amplification [54]. It has thus been suggested that an ideal LAMP detection format would thus include a closed amplification and detection unit to limit contamination [55]. It is recommended to mix reagents and sample on ice to avoid amplification artefacts that can be generated by the polymerase when incubation at a suboptimal temperature (below 45 °C) occurs, as well as keeping the time taken for master mix preparation by less than 30 min [56].

Regarding bovine samples, LAMP detected DNA in tissue samples taken from aborted bovine fetuses from farms having a history of bovine abortion and the presence of dogs. Molecular diagnosis was positive in all cases by both amplification techniques; it is worth stressing that dogs play a main role in *N. caninum* epidemiology because they act as definitive hosts, shedding oocysts in the environment [57], this being a major risk factor for *N. caninum*-associated miscarriages and stillbirths in cattle [58–60]. Nonetheless, this assay’s reliability should be further evaluated through large-scale sampling using clinical samples taken from cows, particularly from herds having a background of abortion or in groups of pregnant cattle and thus ascertain the LAMP method’s ability to detect *N. caninum* DNA.

## Conclusion

A LAMP assay was developed in this study for detecting *N. caninum* DNA based on the parasite’s *Nc-5* gene; the method’s applicability was also evaluated in naturally infected samples. The optimized reaction protocol and conditions described in this study led to a LAMP technique for detecting *N. caninum* DNA, as well as providing a significant contribution; this is the first study reporting *N. caninum* DNA detection by molecular techniques in Colombia. The present LAMP method shows great promise and good prospects regarding its application in fields such as *N. caninum* prevalence monitoring and clinical diagnosis. Future work on validating the method will involve replicating tests to assess the detection limit in different biological samples to determine test performance/reliability in the field.

## Additional files

Additional file 1: Table S1. LAMP test selectivity by in silico analysis using *Nc-5* gene DNA. This file provides information regarding all the sequences found of *Nc-5* gene from different isolates available in databases. (XLSX 16 kb)

Additional file 2: Figure S1. Target region (*N. caninum Nc-5* gene) nucleotide sequence alignment. The annealing site of primers designed for LAMP is shown on the sequences of strains and isolates reported in the GenBank database, as well as percentage identity. (TIFF 12060 kb)

## Abbreviations

95% CI: 95% confidence interval
BLAST: Basic local alignment search tool
LAMP: Loop-mediated isothermal amplification
PCR: Polymerase chain reaction
